# Chemotherapy combined with endocrine neoadjuvant therapy for hormone receptor-positive local advanced breast cancer: a case report and literature review

**DOI:** 10.3389/fendo.2024.1362725

**Published:** 2024-03-13

**Authors:** Nengying Zhang, Chengmin Luo, Jiayang Li, Yuxiang Bao, Zhongliang Yan, Xiaoming Cheng, Taolang Li, Junyuan Lv

**Affiliations:** ^1^ Department of General Surgery, The Affiliated Hospital of Zunyi Medical University, Zunyi, China; ^2^ Department of Breast and Thyroid Surgery, The Second Affiliated Hospital of Zunyi Medical University, Zunyi, China; ^3^ Drug Clinical Trial Institution, The Affiliated Hospital of Zunyi Medical University, Zunyi, China; ^4^ Key Laboratory of Basic Pharmacology of Ministry of Education and Joint International Research Laboratory of Ethnomedicine of Ministry of Education, Zunyi Medical University, Zunyi, China

**Keywords:** breast cancer, chemotherapy, endocrine therapy, capecitabine, fulvestrant

## Abstract

**Background:**

Early studies have revealed antagonistic effects associated with stacking chemotherapy (CT) and endocrine therapy (ET), thereby conventional wisdom does not advocate the simultaneous combination of these two treatment modalities. Limited clinical studies exist on the combined use of neoadjuvant CT (NACT) and neoadjuvant ET (NET), and there are no reported instances of concurrent neoadjuvant treatment for locally advanced breast cancer (LABC) using capecitabine and fulvestrant (FUL).

**Case presentation:**

We reported a 54-year-old woman who was diagnosed with hormone receptor-positive (HR+) LABC at our hospital. After neoadjuvant treatment involving two distinct CT regimens did not lead to tumor regression. Consequently, the patient was transitioned to concurrent capecitabine and FUL therapy. This change resulted in favorable pathological remission without any significant adverse events during treatment.

**Conclusions:**

A novel approach involving concurrent neoadjuvant therapy with CT and endocrine therapy may offer a potentially effective treatment avenue for some cases with HR+ LABC.

## Introduction

The most recent global cancer statistics report reveals that breast cancer has now surpassed lung cancer as the most prevalent malignancy in women for the first time. Notably, it has become the leading cause of cancer-related mortality among women (1). Moreover, the proportion of patients with hormone receptor-positive (HR+) and HER-2 negative (HER2-) BC is notably high, ranging from 65% to 70% (2). Both CT and ET play pivotal roles in the systemic treatment of BC (3, 4). Neoadjuvant therapy for LABC aims to downstage inoperable patients to an operable state and to tailor subsequent treatment strategies through *in vivo* drug sensitization. Numerous subsequent studies have indicated that achieving a pathologic complete response (pCR) during the neoadjuvant stage of BC correlates with a more favorable clinical prognosis. However, for HR+ BC patients (5), the postoperative pCR rate following NACT is relatively low at 18.6%, significantly less effective than that observed for triple-negative and HER-2 overexpressing BCs. In contrast, the pCR rate for NET stands at 3.4%, markedly lower than that of NACT (6). Despite this, all major guidelines recommend NACT as the preferred first-line treatment option over NET for HR+ LABC. Early studies have revealed antagonistic effects associated with stacking CT and ET drugs (7), conventional wisdom does not advocate the simultaneous combination of these two treatment modalities (8–12). Limited clinical studies exist on the combined use of NACT and NET, and there are no reported instances of concurrent neoadjuvant treatment for LABC using capecitabine and FUL. Capecitabine is an oral fluorouracil prodrug with low toxicity and well tolerated; FUL is a selective estrogen receptor down-regulator used primarily in HR+ breast cancer and is easily administered subcutaneously. In this article, we reviewed a case involving the treatment of HR+ LABC with capecitabine in combination with FUL, and summarized the clinical literature on capecitabine in combination with FUL in the treatment of HR+ BC. We aimed to lay a foundation for the clinical use of these drugs in the context of BC.

## Case presentation

A 54-year-old postmenopausal woman was admitted to the hospital due to the discovery of a left breast mass that had been present for three months. Upon physical examination, a 4cm × 4cm mass was identified within the left breast’s upper lateral quadrant near the axilla, along with multiple enlarged lymph nodes in the left axilla alongside the left supraclavicular region. The maximum lymph node measured approximately 2cm × 2cm and exhibited poorly defined borders and limited mobility. No abnormalities were detected in the right breast, axilla, or right supraclavicular region. A breast ultrasound revealed multiple nodules within the left breast’s upper quadrant near the axilla, with the largest nodule measuring 33mm × 15mm, displaying hypoechoic features and unclear borders. Enlarged lymph nodes were also observed in the left axilla, upper and lower clavicle, with sizes of 13mm × 8mm in the axilla, 15mm × 8mm in the left supraclavicular area, and 14mm × 8mm in the left infraclavicular region. These nodules exhibited full morphology, with unclear dermatomedullary demarcation, raising concerns about metastasis. The patient’s breast mammogram at admission is presented in **Supplementary Figure 1**. Subsequent percutaneous needle biopsy of the mass in the left breast confirmed the presence of invasive ductal carcinoma, grade I, estrogen receptor (ER, +, 60%), progesterone receptor (PR, -), HER2 (1+), Ki-67 (45%). Furthermore, the patient with pathologically confirmed left supraclavicular lymph node and left axillary lymph node metastases via puncture biopsy. The detection of serum tumor markers showed elevated levels of CA153 at 403.1 U/mL and CA125 at 59.2 U/mL. Additional tests including thoracic and abdominal CT scans, bone visualization (ECT), and cranial MRI revealed no evidence of metastases. The final diagnosis was invasive ductal carcinoma of the left breast, grade I, cT2N3M0 stage IIIC, Luminal B subtype (HR+/HER2-).

**Figure 1:**
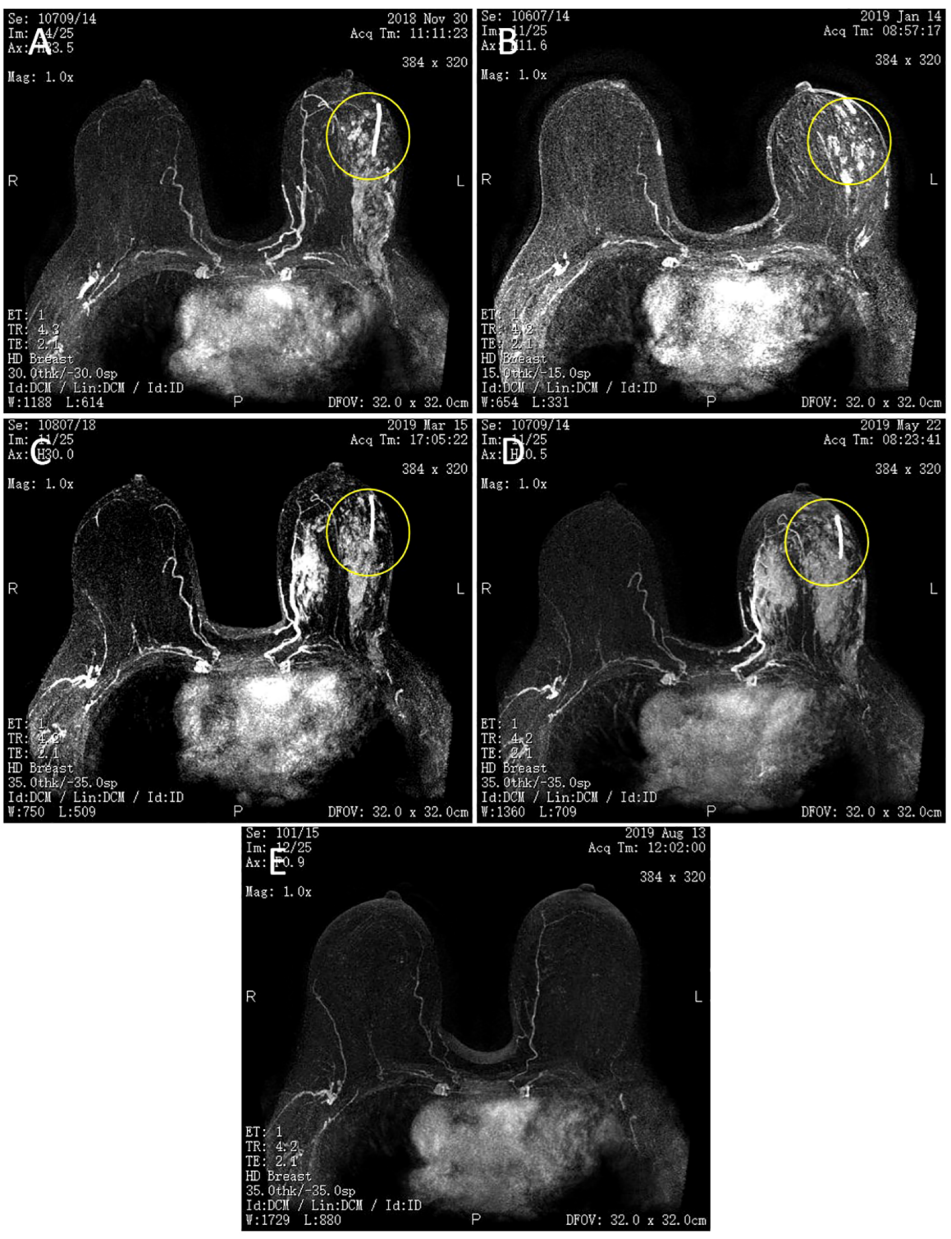
Magnetic resonance imaging (MRI) of bilateral breasts. **(A)** Two cycles of TEC. **(B)** Four cycles of TEC. **(C)** Two cycles of NP. **(D)** Four cycles of NP. **(E)** Capecitabine + FUL for 3 + months. The left breast lesion has disappeared. The yellow circle shows that left breast lesion.

**Figure 2:**
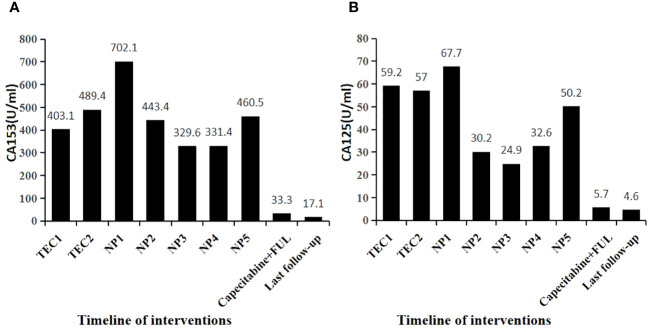
Changes in tumor markers during patient treatment. **(A)** Capecitabine+ FUL for 3 + months. CA153 decreased to below the normal reference value (30U/ml) **(B)** Capecitabine+ FUL for 3+ months. CA125 decreased to below the normal reference value (35U/ml).

**Figure 3:**
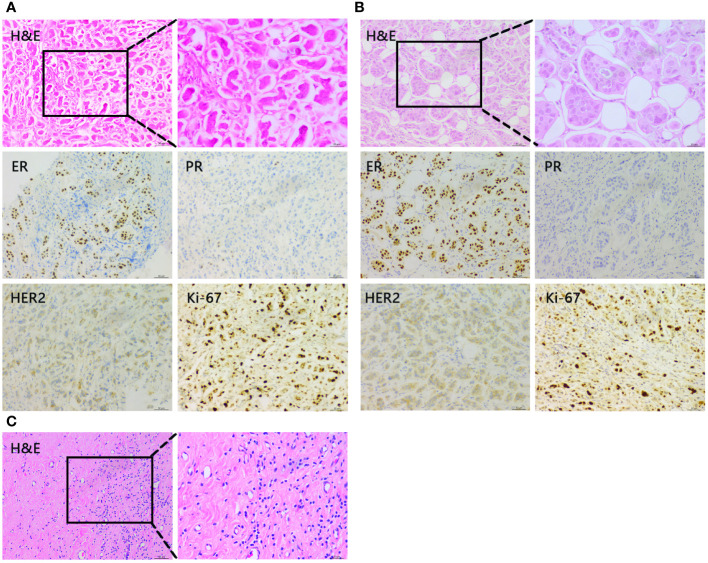
Representative histopathological findings of the primary lesion in the left breast. **(A)** Core needle biopsy of the primary lesion before neoadjuvant chemotherapy. **(B)** Core needle biopsy of the primary lesion after 4 cycles of TEC. **(C)** Tumor of the resected left breast after capecitabine + FUL 3 months.

**Figure 4:**
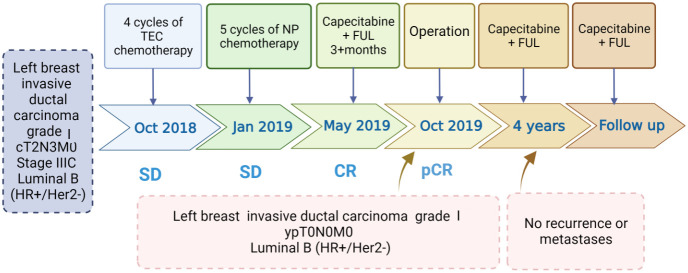
Timeline of interventions and efficacy evaluations. TEC, docetaxel + doxorubicin liposomes+ cyclophosphamide; NP, vinorelbine+ carboplatin; Capecitabine + FUL, capecitabine + fulvestrant.

Following the guidelines outlined by the Expert Consensus on Neoadjuvant Therapy for Breast Cancer in China (2017 edition) (13), the patient initially underwent neoadjuvant therapy with the TEC regimen (docetaxel 130mg + doxorubicin liposomal 40mg + cyclophosphamide 800mg) in October 2018. Upon clinical efficacy assessment using Response Evaluation Criteria In Solid Tumors (RECIST) version 1.1 (14), the evaluation indicated stable disease after 4 cycles. A repeat core needle biopsy of the primary focus was performed and the pathology showed grade I invasive ductal carcinoma of the left breast, ER (+, 60%), PR (–), Her2 (1-2+), Ki-67 (40%). Subsequently, the treatment regimen was switched to the NP regimen (vincristine 40mg + carboplatin 500mg), commencing in January 2019. Despite completing 5 rounds of NP, the lesions exhibited minimal change, and the efficacy assessment confirmed sustained stable disease alongside grade III myelosuppression. Due to the patient’s poor response to the two different CT regimens and her inability to tolerate continued CT, a consensus was reached within the department, leading to a decision to initiate treatment with capecitabine in combination with FUL in May 2019. The prescribed regimen included oral administration of capecitabine 1000 mg/m^2^ twice daily for 2 weeks followed by a 1-week break; along with intramuscular injection of FUL 500 mg once every 2 weeks for the first month, then once every 4 weeks thereafter. Notably, following this modified treatment approach, the patient exhibited a reduction in the size of primary breast foci as well as lymph nodes (**Figure 1**), accompanied by a normalization of serum tumor marker levels (**Figure 2**). Subsequent tests and examinations indicated normal results, culminating in a clinical efficacy assessment confirming complete remission (CR) after 3 months. On October 6, 2019, the patient underwent modified radical surgery for left breast carcinoma. Postoperative pathological findings revealed invasive ductal carcinoma of the left breast, graded as 5 according to the Miller-Payne subsystem. Importantly, no residual tumors were observed upon extensive sampling, and there was no evidence of tumor metastasis in the left axillary lymph nodes (0/13), with the pectoral interstitial lymph nodes exhibiting adipose fibrous connective tissue without tumor involvement (**Figure 3**). Consequently, the patient achieved pCR with the final diagnosis indicating invasive ductal carcinoma of the left breast, grade I ypT0N0M0, Luminal B type (HR+/HER2-). As the patient had achieved pCR, we advised the patient to follow up with endocrine therapy with fulvestrant alone; however, the patient still requested capecitabine in combination with FUL due to concerns about tumor recurrence, in addition to the fact that there were no significant adverse events (AEs) from taking capecitabine in combination with FUL. This patient remains free of the disease 4 years after operation. The treatment flow is illustrated in **Figure 4**.

## Discussion

Both CT and ET are integral components of systemic treatment for HR+ BC. Historically, based on early evidence-based medicine, sequential administration of CT and ET was recommended for HR+ BC patients in various treatment settings, including neoadjuvant, adjuvant, or rescue therapy. This sequential approach was suggested rather than concurrent use due to concerns about potential antagonistic effects between the two modalities. Nevertheless, as our comprehension of tumor biology has advanced and new therapeutic agents have emerged, the concept of concurrent treatment with CT and ET is gaining attention. This shift is further fueled by the development of new drugs as well as the evolution of therapeutic modalities. The conventional practice of sequential therapy may not fully exploit the potential synergistic benefits that could arise from concurrent treatment with CT and ET. In neoadjuvant settings, NACT is considered the standard of care for HR+/HER2- LABC. Conversely, NET achieves both tumor (T) and nodal (N) downstaging within the same subtype (15). Notably, studies have shown that NET can yield overall response rates (ORR) ranging between 20% and 70% with a duration of 3 to 4 months (16).

Capecitabine, an oral prodrug of fluorouracil, undergoes *in vivo* conversion to cytotoxic fluorouracil by carboxylesterase, cytidine deaminase, and thymidylate phosphorylase. It is frequently utilized as a second-line monotherapy for patients resistant to anthracyclines, paclitaxel, or both, demonstrating efficacy in those with metastatic BC (MBC). Notably, findings from the CREATE-X study (17) indicated that incorporating capecitabine adjuvant therapy following standard NACT containing anthracycline, taxane, or both can extend disease-free survival (DFS) alongside overall survival (OS) in HER2-negative BC patients with residual invasive disease on pathological testing. Moreover, Phase II METEORA trial (18) revealed a significantly prolonged time to failure (TTF) and PFS for the oral regimen involving vinorelbine + cyclophosphamide + capecitabine (VEX) compared to intravenous paclitaxel in HR+/HER2- MBC patients. Specifically, the median TTF was reported as 8.3 months vs 5.7 months, and the median PFS (mPFS) was 11.1 months vs 6.9 months. In another study, Rashad et al. (19) assessed ER+/HER2- MBC patients administered with either tamoxifen/capecitabine or letrozole/capecitabine as first-line treatment, demonstrating a 60% ORR and an 82.5% clinical benefit rate (CBR) with the concurrent use of these drug combinations. However, the application of capecitabine in NACT for BC has not been extensively documented in existing literature.

FUL is a selective ER down-regulator that effectively prevents endogenous estrogen from binding to the ER. This action obstructs ER dimerization as well as DNA binding, enhances ER turnover to impede the receptor’s nuclear uptake, and promotes proteasomal degradation, consequently inhibiting tumor cell proliferation (20). Approved for HR+ advanced BC and postmenopausal women experiencing disease progression post anti-estrogen therapy (21). Wang et al. (22) demonstrated that FUL in combination with vincristine had a synergistic effect among patients with HR+/HER2- recurrent BC/MBC, with a median follow-up of 25.1 months as well as an overall PFS of 9.86 months, in contrast with a mPFS of 20.73 months and 4.27 months for the first- and second-line treatment populations, respectively, indicating the potential advantage of concurrent use of CT and FUL. Furthermore, multiple studies have highlighted the favorable clinical benefits of FUL during the neoadjuvant phase in HR+ postmenopausal BC. A phase II clinical study by Quenel et al. (23) reported an ORR of 53.8% with FUL neoadjuvant therapy after 6 months in patients with operable or HR+ locally advanced postmenopausal BC. In another study by Lerebours et al. (24) involving HR+/HER2- postmenopausal BC patients, the CBR of FUL neoadjuvant therapy after 6 months was determined to be 36.8%.

Clinical investigations examining the utility of capecitabine in conjunction with FUL have robustly demonstrated that this combined therapy significantly extends patient prognosis while maintaining excellent tolerability. Schwarzberg et al. (25) phase II study meticulously examined the efficacy and toxicity of a treatment regimen integrating ET and low-dose metronomic CT, comprising FUL and capecitabine, for HR+/HER2- MBC. Their findings revealed a mPFS of 14.98 months, a median time to progression (TTP) of 26.94 months, as well as a median OS (mOS) of 28.65 months. Notably, the treatment demonstrated outstanding tolerance, as evidenced by less than 10% incidence of Grade 3 palmar-plantar erythrodysesthesia. Additionally, Anna et al. (26), through retrospective data analysis, investigated ER+/HER2- MBC patients administered with FUL combined with MC (cyclophosphamide, vinorelbine, as well as capecitabine), revealing the mPFS of 8.4 months as well as the mOS of 21.5 months. These findings together demonstrate the safety and effectiveness of combining capecitabine with FUL in managing HR+/HER2- advanced BC. This contrasts previous studies that suggested concurrent treatment with CT and ET compromised treatment efficacy and exacerbated adverse effects, prompting a reevaluation of the optimal regimen and timing for neoadjuvant treatment of HR+/HER2- BC.

In this case of a patient with HR+ LABC, two distinct chemotherapy regimens were utilized before and after the neoadjuvant phase, resulting in poor efficacy evaluation. However, following the replacement of the prior treatment with concurrent administration of capecitabine and FUL for over 3 months, the postoperative pathological response evaluation demonstrated a pCR. Remarkably, the patient experienced no treatment-related AEs during the course and remained free from disease recurrence for over 4 years. This report marks the first instance of employing the “new regimen,” featuring concurrent capecitabine and FUL during the neoadjuvant phase in HR+/HER2- LABC patients. This pioneering approach suggests that the simultaneous use of CT and ET in HR+ BC holds promising clinical value, particularly as capecitabine, an orally administered agent, offers ease of use and a more favorable cost-effectiveness balance compared to traditional intravenous CT drugs.

## Conclusions

The incidence of BC has been rising year by year, underscoring the significance of CT and ET as pivotal modalities for its treatment. Traditionally, the prevailing notion suggested that concurrent administration of these two treatments not only proved ineffective but also amplified drug toxicity, thereby disfavoring their simultaneous use. However, with ongoing exploration of therapeutic approaches and the introduction of new drugs, increasing evidence has demonstrated that combining CT and ET can enhance patient prognosis without introducing more severe toxicity and side effects. As a result, this combined approach has emerged as a viable treatment option for individuals grappling with HR+ LABC and even metastatic cases. This evolving understanding has led to a paradigm shift in the choice of treatment, heralding a new era wherein the synergistic use of CT and ET holds promise for improved patient outcomes with no added detriment in terms of treatment-related AEs.

## Data availability statement

The raw data supporting the conclusions of this article will be made available by the authors, without undue reservation.

## Ethics statement

The studies involving humans were approved by Medical Ethics Committee of Zunyi Medical University. The studies were conducted in accordance with the local legislation and institutional requirements. Written informed consent for participation was not required from the participants or the participants’ legal guardians/next of kin in accordance with the national legislation and institutional requirements. Written informed consent was obtained from the individual(s) for the publication of any potentially identifiable images or data included in this article.

## Author contributions

NZ: Conceptualization, Data curation, Formal analysis, Investigation, Methodology, Software, Visualization, Writing – original draft. CL: Conceptualization, Data curation, Formal analysis, Investigation, Methodology, Software, Visualization, Writing – original draft. JiL: Methodology, Data curation, Formal analysis, Writing – review and editing. YB: Visualization, Writing – original draft, Data curation. ZY: Formal analysis, Writing – original draft. XC: Writing – review and editing, Conceptualization, Investigation. TL: Project administration, Writing – review and editing, Conceptualization, Data curation, Formal analysis, Methodology, Resources, Validation, Investigation, Supervision. JuL: Project administration, Writing – review and editing, Conceptualization, Data curation, Formal analysis, Methodology, Resources, Validation, Investigation, Supervision.

## Supplementary material

Supplementary Figure 1 Molybdenum target examination of the left breast before neoadjuvant chemotherapy. **(A)** High-density dotted mass with an irregular margin. **(B)** High-density Axillary lymph nodes with an irregular margin.
