# Supplementary figures and images for: Chemotherapy combined with endocrine neoadjuvant therapy for hormone receptor-positive local advanced breast cancer: a case report and literature review
Source: Front Endocrinol (Lausanne). 2024 Mar 13;15:1362725. doi: 10.3389/fendo.2024.1362725 (PMC10976348; doi:10.3389/fendo.2024.1362725)

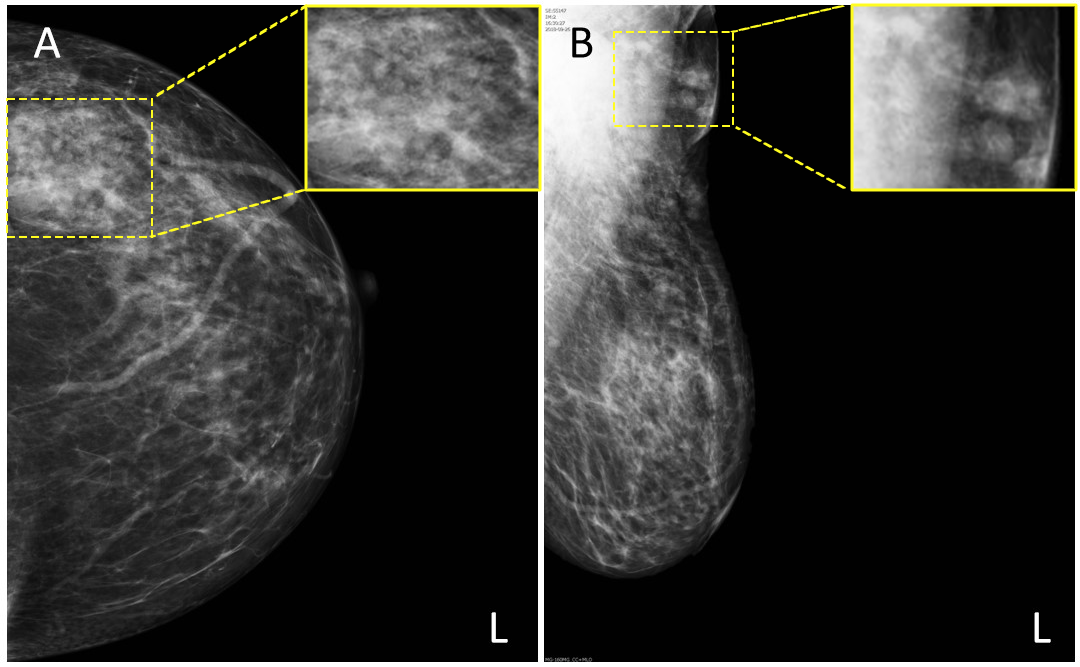

Supplement: Supplementary Figure 1 — Molybdenum target examination of the left breast before neoadjuvant chemotherapy. (A) High-density dotted mass with an irregular margin. (B) High-density Axillary lymph nodes with an irregular margin. [file Image_1.tif]
